# Life-Preservers

**Published:** 1871-09

**Authors:** 


### LIFE-PRESERVERS,

Made of India Rubber, are worse than worthless, because they are so easily punctured, or rendered otherwise unavailable, that many relying upon them perish, who, trusting to their own efforts, might have been saved. Articles made of cork are every way preferable, provided they weigh at least six pounds, as less than that will not sustain an average sized person in the water. It should be remembered and practiced upon by those who swim and those who cannot, that the body may be made to float a long time on the water without much effort, if it is thrown on its back, the hands clasped under, or, better, projected bottomward in the shape of a wedge, while the head is thrown back, the nose only projecting above the water. In this way, also, persons exhausted by swimming may rest themselves.

